# Paternal Leave and Father-Infant Bonding: Findings From the Population-Based Cohort Study DREAM

**DOI:** 10.3389/fpsyg.2021.668028

**Published:** 2021-06-04

**Authors:** Ronja Schaber, Marie Kopp, Anna Zähringer, Judith T. Mack, Victoria Kress, Susan Garthus-Niegel

**Affiliations:** ^1^Institute and Policlinic of Occupational and Social Medicine, Faculty of Medicine Carl Gustav Carus, Technische Universität Dresden, Dresden, Germany; ^2^Department of Medicine, Faculty of Medicine, Medical School Hamburg, Hamburg, Germany; ^3^Department of Child Health and Development, Norwegian Institute of Public Health, Oslo, Norway

**Keywords:** paternal leave, solo paternal leave, childcare, father-infant bonding, partnership satisfaction, mediation analysis, DREAM study

## Abstract

**Background:** Father-infant bonding is important for child development. Yet, in contrast to mother-infant bonding, little is known about factors that might facilitate father-infant bonding. With new generations of fathers being more involved in childcare, this study aims to examine the impact of paternal leave duration on father-infant bonding, and whether this relation is mediated by the amount of time fathers actively spend on childcare.

**Methods:** Data of *n* = 637 fathers were derived from the German population-based cohort study “Dresden Study on Parenting, Work, and Mental Health” (DREAM). Mediation analyses were conducted. Duration of paternal leave (predictor), weekly hours spent on childcare (mediator), and father-infant bonding (outcome) were measured at 14 months postpartum. The potential confounders current status of paternal leave, part-time work during paternal leave, duration of solo paternal leave, age, education, and partnership satisfaction were included in a second mediation analysis.

**Results:** Without considering confounders, duration of paternal leave positively predicted father-infant bonding through weekly hours spent on childcare. When adding confounders to the model, this indirect path did not stay significant. Moreover, in the adjusted model and on the direct path duration of paternal leave negatively predicted father-infant bonding. Additionally, partnership satisfaction positively predicted father-infant bonding. Some study variables were significantly associated with the mediator. Longer duration of paternal leave, currently being on paternal leave, younger age, and lower educational level predicted more weekly hours spent on childcare.

**Conclusions:** Duration of paternal leave not being a stable predictor for father-infant bonding suggests that fathers, who do not have the opportunity to take long periods of paternal leave, can still form strong bonds with their infants. Other factors, for example partnership satisfaction, which might represent fathers' underlying capacity to bond, might be more crucial for father-infant bonding. At the same time, results should not be interpreted in a way that father involvement (e.g., paternal leave/time spent) does not matter for children's development. The finding that longer duration of paternal leave increases weekly hours spent on childcare supports the idea that facilitating father involvement can be achieved by paternal leave incentives such as non-transferable father months.

## Introduction

Parent-infant bonding has been argued to be “the central and most important psychological process of the puerperium” (Brockington et al., 2006, p. 243). Bonding is the parent's emotional tie or love toward their child, not to be confused with parent involvement or children's attachment (Kinsey and Hupcey, 2013). Parent-infant bonding is considered to be the necessary basis for positive parenting behaviors (Condon, 1993; Condon and Corkindale, 1998). It is not surprising thus, that several studies find support for the importance of parent-infant bonding in child development (Yalçin et al., 2010; Mason et al., 2011; Fuchs et al., 2016; de Cock et al., 2017). Therefore, research on factors that can strengthen parent-infant bonding is needed. While factors promoting or hindering mother-infant bonding have been researched to some extent (for overview, see Kinsey and Hupcey, 2013), much less is known about factors predicting father-infant bonding (Scism and Cobb, 2017).

In his process model of parenting, Belsky (1984) suggested three domains of competent parenting, which might also influence the father-infant bond: personal psychological resources of parents, contextual sources of stress and support, and characteristics of the child. The newly emerging body of literature on father-infant bonding has already identified some factors associated with father-infant bonding which can be integrated into the domains of the model (as previously done by de Cock et al., 2016; Wynter et al., 2016). Concerning **fathers' characteristics** (i.e., personal resources), being a younger father (Hall et al., 2015) and having a lower educational level (Hall et al., 2015; de Cock et al., 2016) were associated with higher bonding. Regarding personality traits, higher levels of extraversion, conscientiousness, agreeableness, and emotional stability were all associated with higher levels of father-infant bonding (de Cock et al., 2016). Moreover, having lower levels of personality traits entailing a vulnerability to postnatal depression (e.g., sensitivity to the opinions of others), was associated with higher levels of father-infant bonding (Wynter et al., 2016). Fathers' perceived care by their own parents also influenced father-infant bonding positively (Hall et al., 2015). Depression in fathers was associated with lower father-infant bonding in multiple studies (Parfitt et al., 2014; Kerstis et al., 2016; Wynter et al., 2016; Nishigori et al., 2020). Concerning **contextual factors**, partner support and quality of relationship were positively associated with father-infant bonding in multiple studies (Condon et al., 2013; de Cock et al., 2016; Kerstis et al., 2016; Wynter et al., 2016; Nishigori et al., 2020), only Parfitt et al. (2014) found some mixed results at different measurement points. In couples, higher levels of mother-infant bonding were associated with higher levels of father-infant bonding (Nishigori et al., 2020), and depression in mothers was associated with lower father-infant bonding (Kerstis et al., 2016). Parenting stress was associated with lower levels of bonding (de Cock et al., 2016), which seems consistent with the finding that a difficult **child** temperament was also associated with lower levels of bonding (Condon et al., 2013; Parfitt et al., 2014; de Cock et al., 2016). Concerning parity, de Cock et al. (2016) found higher bonding levels in primiparous fathers. Multiple studies show that father-infant bonding levels stay relatively stable over different measurement points (Condon et al., 2013; Parfitt et al., 2014; Hall et al., 2015; de Cock et al., 2016).

Since Belsky (1984) has developed the model of competent parenting, an important new contextual aspect of fathering has emerged. Fathers are becoming more and more involved in child-care and parental leave reforms with special incentives for fathers to stay at home are being passed in OECD countries (Castro-García and Pazos-Moran, 2016; Gauthier and Bartova, 2018). Following the last parental leave reform in Germany, every mother and father has the right to take parental leave for a maximum of 3 years. Of these 3 years, the couples can receive parental allowance for 12 months, which can be stretched to 14 months if both parents undertake at least 2 months (non-transferable partner months; BMFSFJ, 2020). Since the reform, the proportion of fathers taking paternal leave has risen, even though slowly and from a very low level, but steadily (Samtleben et al., 2019; Statistisches Bundesamt [Desatis], 2020). Despite these current social developments, it has not been researched how paternal leave and spending time with the child influence father-infant bonding. The present study aims to close this gap in the literature.

Even though, to the best of our knowledge, the association between time and father-infant bonding has not been researched before, there are some indications in the literature, that spending (more) time with the newborn may foster father-infant bonding. One explanation for higher levels of bonding in primiparous fathers could be a greater amount of time spent with a single child in comparison to fathers whose available time has to be divided between multiple children (de Cock et al., 2016). This idea is supported by the finding that mother-infant bonding levels were higher in comparison to father-infant bonding (Hall et al., 2015; de Cock et al., 2016), which may indicate that time is an important factor, as mothers typically spend more time with their infant than fathers. Fathers themselves seem to believe that spending sufficient time with a child is an indispensable factor for forging an intimate bond (Brady et al., 2016). Moreover, children whose fathers took paternal leave (Petts et al., 2020) and were more involved in childcare until age one (Jessee and Adamsons, 2018) report better father-child relationships at age nine compared to children who experienced less father involvement during their 1st year of life. On the basis of these indications, we hypothesize that spending (more) time with the newborn will positively influence father-infant bonding.

Taking paternal leave and spending time with the child are closely related. According to an explorative German survey, fathers who have been intensively involved in childcare and family activities during the child's 1st months of life intend to continue their active involvement in the family to maintain the intimate relationship with their child (Pfahl and Reuyss, 2010). In fact, one quarter of German fathers who took paternal leave (vs. who did not) were shown to have reduced their working hours after their paternal leave (Hobler and Pfahl, 2015). Furthermore, German fathers who took paternal leave had a higher involvement in childcare tasks after their paternal leave ended (Bünning, 2015). Similar results of fathers who took paternal leave being more involved in childcare activities later have been found in U.S., U.K., and Spanish populations (Tanaka and Waldfogel, 2007; Romero-Balsas, 2015; Pragg and Knoester, 2017). Concerning the duration of paternal leave, studies are more inconsistent. While some studies could not find an association between duration of paternal leave and involvement in childcare (Bünning, 2015), others found that longer periods of paternal leave result in higher levels of childcare involvement (Pragg and Knoester, 2017). On the basis of these indications, we hypothesize that taking paternal leave (irrelevant of duration) and in some cases taking longer periods of paternal leave increases the amount of time fathers spend on childcare later.

Combining these results, we hypothesize that spending more time with the child may predict higher levels of father-infant bonding. Fathers have the opportunity to spend time with their children during paternal leave, which in turn might influence the number of hours spent with the child after paternal leave. Therefore, we hypothesize a mediated relationship between *X* (duration of paternal leave) and *Y* (father-infant bonding) through *M* (weekly hours spent on childcare).

When researching this relation, some specifications of paternal leave have to be considered as confounding factors, including current status of paternal leave, part-time work during paternal leave, and solo paternal leave. If the father is currently on paternal leave, he will most likely spend more hours actively engaging with his child in comparison to if he is not on paternal leave and has a standard full-time workday of 8 h, during which he commonly does not spend the majority of the day with his child. Concerning part-time work during paternal leave, in Germany it is possible to work up to 30 h while being on paternal leave and receiving parental allowance (BMFSFJ, 2020). Fathers choosing this option will have less opportunity to spend time with their children. Taking solo paternal leave lies at the other end of the spectrum: Some parents decide to stagger parental leave. Taking solo paternal leave could give fathers more opportunity to actively engage with their children, as the mother will commonly not be at home during her work hours. The construct of solo paternal leave is still fairly unexplored. While Bünning (2015) did not find solo paternal leave to have a significant additional influence on time spent on childcare after the end of paternal leave, data from an Australian qualitative study indicate that solo caring fathers feel more attached and close to their children than fathers who did not take solo paternal leave (Wilson and Prior, 2010).

Further confounders that might influence the postulated mediation are fathers' age, education, and partnership satisfaction. All three have previously been shown to be related to father-infant bonding (see paragraph 2; Condon et al., 2013; Hall et al., 2015; de Cock et al., 2016; Kerstis et al., 2016; Wynter et al., 2016; Nishigori et al., 2020) and they might also be associated to duration of paternal leave and hours spent on childcare. Younger German men agree more often than older men that fathers should reduce their work while their children are small (Wippermann, 2017), and new generations of German fathers wish to be more involved in childcare than previous ones (Juncke et al., 2018). Regarding education, a higher educational level was associated with taking longer periods of parental leave in some studies (Lappegard, 2008), while others yielded mixed results (Sundström and Duvander, 2002) or no association at all (Geisler and Kreyenfeld, 2011). Partnership satisfaction 9 months postpartum is positively associated with paternal leave (Petts and Knoester, 2019). In addition, irrespective of whether paternal leave is taken or not, fathers' active involvement in the 1st years of parenthood is positively associated with relationship quality (McClain and Brown, 2017).

**Figure 1:**
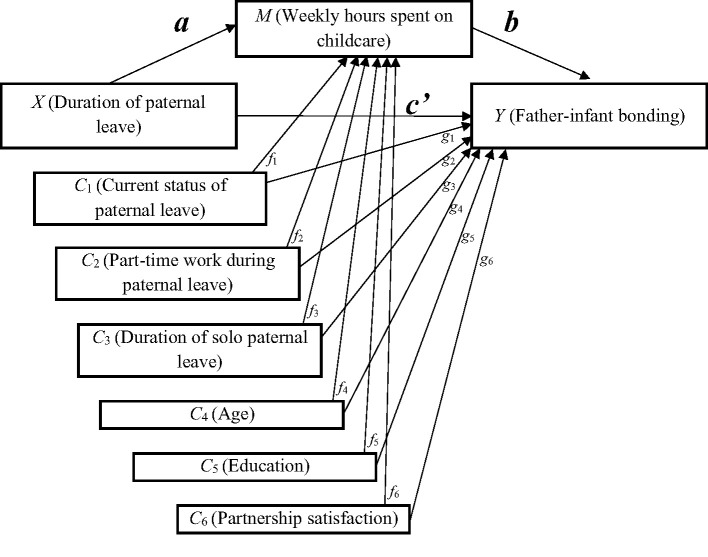
The hypothesized association between duration of paternal leave and father-infant bonding through weekly hours spent on childcare, including six potential confounders. *X*, predictor variable; *M*, mediator variable; *Y*, outcome variable; *C*_1−6_, confounders; *a*, effect of *X* on *M*; *b*, effect of *M* on *Y*; *ab*, indirect effect of *X* on *Y*; *c'*, direct effect of *X* on *Y*, estimates the difference between *X* and *Y* holding *M* constant; *f*_1−6_, effects of *C*_1−6_ on *M*; *g*_1−6_, effects of *C*_1−6_ on *Y*.

Contrary to most previous literature in the field researching mother-infant bonding, the present study focuses on factors potentially related to father-infant bonding. A positive relation between duration of paternal leave and father-infant bonding at the child's age of 14 months is assumed. Further, it is hypothesized that this relation is mediated by the time fathers actively spend on childcare. Potential confounders, i.e., current status of paternal leave, part-time work during paternal leave, solo paternal leave, age, education, and partnership satisfaction are included (Figure 1).

## Materials and Methods

### Study Design and Sample

The present study is part of the Dresden Study on Parenting, Work, and Mental Health (“**DR**esdner Studie zu **E**lternschaft, **A**rbeit und **M**entaler Gesundheit,” **DREAM**), a longitudinal multi-method cohort study of a community sample. Expectant mothers and their partners were recruited during pregnancy mostly at information events of obstetrical clinics and birth preparation courses in and around the city of Dresden, Germany. The aim of the DREAM study is “to prospectively investigate the relationship between parental work participation, role distribution, stress factors, and their effects on perinatal outcomes and long-term family mental and somatic health […]” (Kress et al., 2019, p. 1). Participants complete various questionnaires at six measurement points: during pregnancy (T1), 8 weeks after the anticipated birth date (T2), 14 months (T3), 2 years (T4), 3 years (T5), and 4.5 years (T6) after the actual birth date. Further details regarding the study design of DREAM are described in the study protocol (Kress et al., 2019).

**Figure 2:**
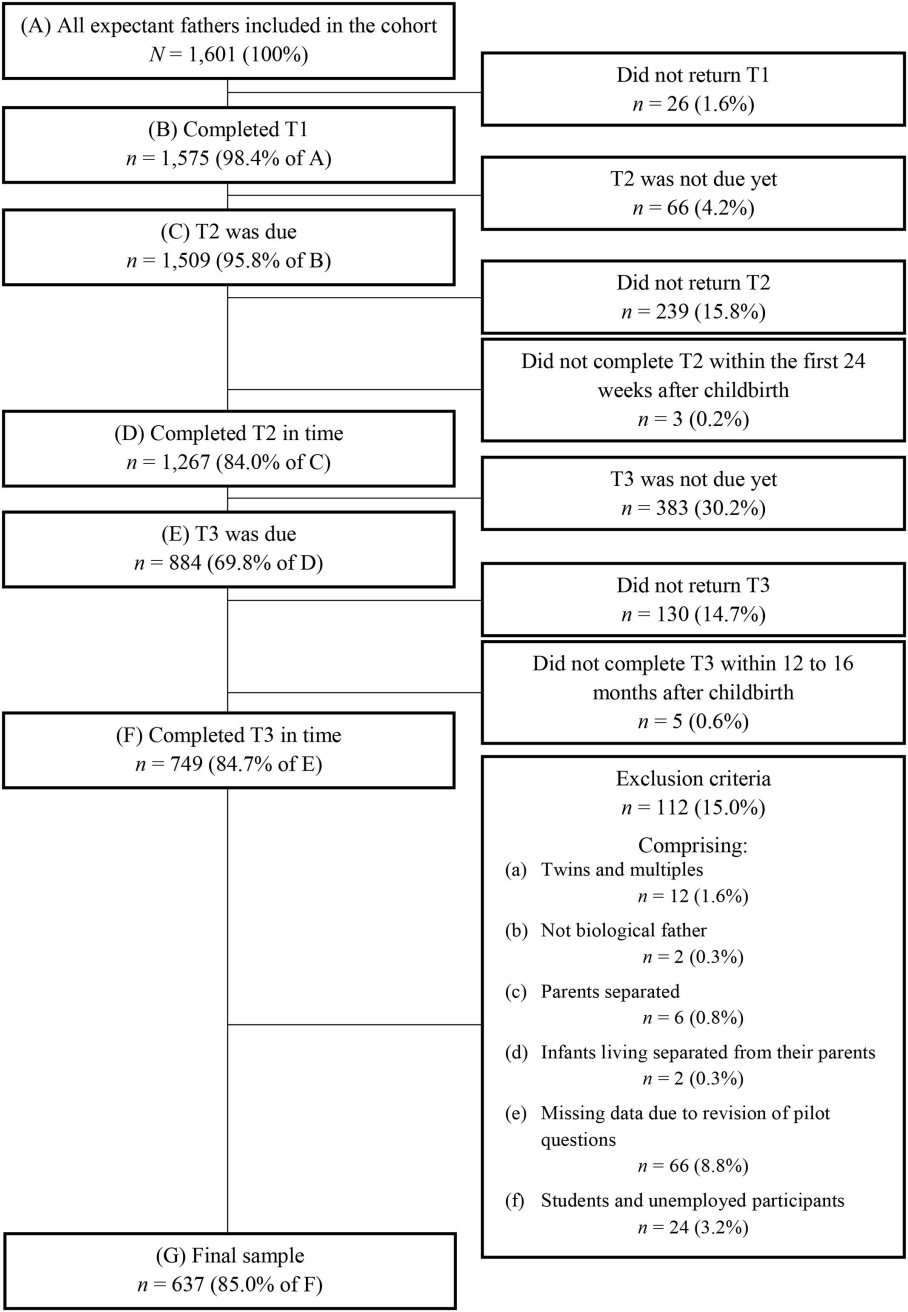
Flowchart of retention rate and exclusion criteria resulting in final sample. T1, during pregnancy; T2, around 8 weeks after anticipated birth date; T3, around 14 months after actual birth date. Data from 3^rd^ of December 2020 (prospective data collection ongoing).

The present paper investigates data from participating fathers having completed T1, T2, and T3. As presented in Figure 2, the number of eligible participants for the present study consists of *N* = 1, 601 expectant fathers of which *n* = 1, 575 had completed the T1 questionnaire at the time of data extraction on the 3^rd^ of December 2020 (prospective data collection ongoing). Inclusion criteria were the timely completion of T2 and T3. Further, *n* = 22 (3.0%) participants were excluded due to factors such as having had twins or multiples, not being the biological father, parents being separated, and infants living separated from their parents, all measured at T3. Some T3 questions relevant for the present study had to be revised after the pilot phase, therefore *n* = 66 (8.8%) participants who had answered the first version of questions were excluded. Further, as this study investigates the duration of paternal leave, *n* = 24 (3.2%) students and unemployed participants who had not been entitled to parental leave were excluded at T3. Exclusion criteria did not entail any health measures, as we aimed to leave the sample as diverse as possible, to be able to generalize the results to the community. The final sample consisted of *n* = 637 fathers.

### Instruments

Study data were collected and managed using Research Electronic Data Capture (REDCap), a secure, web-based software platform designed to support data capture for research studies, hosted at “Koordinierungszentrum für Klinische Studien” at the Faculty of Medicine of the Technische Universität Dresden (Harris et al., 2009, 2019).

**Father-infant bonding** was measured at T3 with the validated German version of the Postpartum Bonding Questionnaire (PBQ; Brockington et al., 2001; Reck et al., 2006), a self-rating instrument consisting of 25 items. The participants are instructed to think about the most difficult time with their child and rate the items (e.g., “I feel angry with my baby” or “I feel close to my baby” [reversed]) on a 6-point Likert scale ranging from 0 (*never*) to 5 (*always*). In the original version of the PBQ, higher scores (ranging from 0 to 125) indicate more bonding problems. For the present study, the items were reversed so that higher scores indicate a higher level of father-infant bonding. Therefore, in the presented data, a score of 99 or less is the clinical cut-off point for the identification of a possible bonding disorder (Brockington et al., 2006). In the present study the reliability of the PBQ was excellent (Cronbach's α = 0.86).

**Duration of paternal leave** is the sum duration (number of months) of all fathers' self-reported paternal leave periods from childbirth up to the date of completing the questionnaire, around 14 months postpartum. At T3, fathers answered retrospective questions about their parental leave (own and partner's) including duration, number of periods, beginning, and end of parental leave periods.

**Weekly hours spent on childcare** represents the number of hours per week, fathers actively engage in childcare activities such as feeding, putting to bed, dressing, organizing chaperones, playing, or talking at T3. To estimate the weekly hours, several questions based on the 1997 National Study of the Changing Workforce were used (Hall and MacDermid, 2009). The first item used in the present study aims to detect fathers' average number of days of gainful employment per week. Subsequently, fathers had to estimate how many hours per day they spend on childcare activities on (a) a day of gainful employment (workday) and (b) a day without gainful employment (work-free day). If participants were currently on paternal leave, they only answered part b. By multiplying the hours spend on childcare with the corresponding number of work- and work-free days, the number of hours per week was calculated.

**Current status of paternal leave** was assessed by fathers' self-report, with 0 indicating fathers have *never been or already finished paternal leave* and 1 indicating fathers are *currently on paternal leave* at T3.

**Part-time work during paternal leave** was assessed by fathers' self-report at T3, with 0 indicating *no part-time work during paternal leave* and 1 indicating *part-time work during paternal leave*.

**Duration of solo paternal leave** is the sum of all paternal leave periods in months that the father took on his own, i.e., not parallel to maternal leave. In other words, the number of months the father stayed at home with the child, while the mother was working. Duration of solo paternal leave was, as duration of paternal leave, determined by the retrospective questions about the fathers' own and their partners' parental leave periods until T3.

**Age and education** were measured at T1. Age was measured in years. Education was measured with the question “Which vocational training qualifications do you have?” based on the German National Cohort Consortium (2014). Answers were categorized into 0 (*no university degree*) and 1 (*university degree*).

**Partnership satisfaction** was measured at T2 using the validated German short version of the Partnership Questionnaire (PFB-K; Kliem et al., 2012). The PFB-K is a self-rating instrument consisting of nine items (e.g., “We talk to each other for at least half an hour in the evening” or “She blames me when something has gone wrong” [reversed]). Response categories range from 0 (*never/very rare*) to 3 (*very often*) with a sum score of 27 indicating the highest level of partnership satisfaction. In the present study the reliability of the PFB-K was good (Cronbach's α = 0.77).

### Statistical Analyses

All statistical analyses were conducted by using IBM SPSS Statistics 26 (IBM Corp, 2019). In case of missing values for items of a sum score, those were substituted with the participant's mean value if no more than 20% of items were missing on this scale. Before conducting descriptive analyses, non-plausible values were set to missing (e.g., if participants claimed to work more than 7 days per week or to spend more than 16 h per day on childcare activities such as feeding, putting to bed, dressing, organizing chaperones, playing, or talking). Before conducting the main analyses, outliers and extreme values outside of the bounds [Q_1_−1.5 ^*^ IQR; Q_3_ + 1.5 ^*^ IQR] were excluded. After exclusion of outliers and extreme values, the main assumptions of the linear model, including linearity, independent errors, homoscedasticity, normally distributed errors, and no multicollinearity were tested and could be confirmed (Hayes, 2018). Due to the exclusion of outliers and extreme values and some missing data, *n* varied between the different analyses.

To acquire information on the sociodemographic characteristics of the sample and all study variables, descriptive data analyses were carried out. The relationships between predictors, confounders, and outcome were examined by using Pearson's correlation. To investigate the postulated mediation (*X*, duration of paternal leave on *Y*, father-infant bonding through *M*, weekly hours spent on childcare) two simple mediation analyses (first without, second with consideration of six potential confounders) were carried out using the SPSS modeling tool PROCESS v3.5 macro by Hayes (2018). The tool uses ordinary least squares regression, yielding unstandardized path coefficients for total (*c*), direct (*c'*), and indirect effects (*ab*). For the present mediation, heteroscedasticity consistent standard errors (HC3) according to Davidson and MacKinnon (1993) were employed. BCa-Bootstrapping with 5,000 samples was applied to compute the confidence intervals and inferential statistics. Effects are significant if the confidence interval does not include zero (Hayes, 2018). To have an orientation concerning the power of the mediation, the simulation-based calculations of Fritz and MacKinnon (2007) were considered. For the individual regression models of the mediation, *post hoc* power analyses were conducted with G^*^Power 3 (Faul et al., 2007).

### Ethical Statement

The DREAM study was approved by the Ethics Committee of the Faculty of Medicine of the Technische Universität Dresden (No: EK 278062015). All participants received written information about the aims and procedures of the study during recruitment. They were informed about pseudonymization of their data and their right to withdraw from the study at any time. All participants signed a declaration of consent.

## Results

### Descriptive Statistics

The characteristics of the final sample are presented in Table 1. The majority of participants were born in Germany (98.0%, *n* = 622), had a university degree (57.5%, *n* = 362), and a full-time employment (77.0%, *n* = 466) at measurement point T1. The duration of paternal leave, fathers had taken until T3, ranged from 0 to 15 months (*M* = 2.4, *SD* = 2.4). A possible bonding disorder with bonding scores below the clinical cut-off point (Brockington et al., 2006) was identified for 7.6% (*n* = 46) of fathers. Intercorrelations between all study variables are presented in Table 2. The largest correlation was between duration of solo paternal leave and current status of paternal leave [*r*_(472)_ = 0.33, *p* < 0.001], meaning that there were no large correlation coefficients, i.e., *r* ≥ 0.5 between the study variables.

**Table 1:** Sample description.

**Sample characteristics**	**Total (*****n*** **=** **637)**	
	***n*^**a**^ (%^**b**^)**	**M ± SD (range)**
Age in years (T1)		32.3 ± 4.7 (20–48)
Week of partners pregnancy (T1)		30.6 ± 6.2 (8–41)
**Country of birth (T1)**		
Germany	622 (98.0)	
Other	13 (2.0)	
**Education (T1)**		
No university degree	268 (42.5)	
University degree	362 (57.5)	
**Parity (T1)**		
Primiparous	486 (77.9)	
Multiparous	138 (22.1)	
**Employment status (T1)**^**c**^		
Full-time employed	545 (85.8)	
Part-time employed	52 (8.2)	
Marginally employed	15 (2.4)	
Others^d^	56 (8.8)	
Infant age in weeks (T2)		9.1 ± 2.3 (4–21)
Partnership satisfaction (T2; 0–27)^e^		19.8 ± 4.0 (6–27)
Infant age in months (T3)		13.9 ± 0.5 (12–16)
**Employment status (T3)**^**c**^		
Full-time employed	466 (77.0)	
Part-time employed	95 (15.7)	
Marginally employed	9 (1.5)	
Others^f^	0 (0)	
Father-infant bonding (T3; 0–125)^g^		111.9 ± 8.1 (81–125)
Duration of paternal leave in months (T3)^h^		2.4 ± 2.4 (0–15)
Weekly hours spent on childcare (T3)		28.0 ± 13.1 (5–112)
**Current status of paternal leave (T3)**		
Never been or already finished paternal leave	504 (82.4)	
Currently on paternal leave	108 (17.6)	
**Part-time work during paternal leave (T3)**		
No part-time work during paternal leave	422 (83.2)	
Part-time work during paternal leave	85 (16.8)	
Duration of solo paternal leave in months (T3)^i^		0.7 ± 1.5 (0–12)

*T1, Measurement point during pregnancy; T2, Measurement point around 8 weeks after the anticipated birth date; T3, Measurement point around 14 months after the actual birth date*.

a *n slightly varies due to missing data of some participants*.

b *Valid percent*.

c *Multiple answers allowed*.

d *Including irregular employment, apprenticeship, student, unemployed, and others*.

e *Short version of the Partnership Questionnaire (“Kurzform des Partnerschaftsfragebogens”, PFB-K)*.

f *Including irregular employment, apprenticeship, and others (not including students and unemployed participants who were excluded as they are not entitled to parental leave)*.

g *Postpartum Bonding Questionnaire (PBQ); reversed items so that higher scores indicate a higher level of father-infant bonding*.

h *Sum of all paternal leave periods until T3*.

i *Sum of all paternal leave periods that the father took on his own, i.e., not parallel to maternal leave, until T3*.

**Table 2:** Intercorrelations between study variables.

	**1**	**2**	**3**	**4**	**5**	**6**	**7**	**8**	**9**
1. Father-infant bonding^a^	—								
2. Duration of paternal leave^b^	−0.08	—							
3. Weekly hours spent on childcare	0.11^*^	0.12^**^	—						
4. Current status of paternal leave	0.02	0.07	0.22^**^	—					
5. Part-time work during paternal leave	−0.10	0.12^*^	0.16^**^	0.26^**^	—				
6. Duration of solo paternal leave^c^	0.03	0.27^**^	0.18^**^	0.33^**^	0.06	—			
7. Age	0.03	0.02	−0.17^**^	−0.11^*^	−0.09	−0.13^**^	—		
8. Education	−0.05	0.16^**^	−0.07	0.06	0.03	0.05	0.06	—	
9. Partnership satisfaction^d^	0.14^**^	0.08	0.05	0.08	0.01	0.04	−0.03	0.08	—

*Specification of the Pearson's correlation coefficient r. Two-tailed. Outliers and extreme values excluded, n varies between 385 and 474 due to missing data of some participants*.

a *Postpartum Bonding Questionnaire (PBQ); reversed items so that higher scores indicate a higher level of father-infant bonding*.

b *Sum of all paternal leave periods in months until T3*.

c *Sum of all paternal leave periods in months that the father took on his own, i.e., not parallel to maternal leave until T3*.

d *Short version of the Partnership Questionnaire (“Kurzform des Partnerschaftsfragebogens”, PFB-K)*.

* *p < 0.05*.

** *p < 0.01*.

### Dropout Analyses

Dropout analyses were conducted for sociodemographic characteristics and partnership satisfaction of the completer group vs. the non-completer group. Completers were more often born in Germany (98.0 vs. 95.1%; Fisher's exact test, *p* = 0.025), and more often had a university degree [57.5% vs. 39.3%; χ^2^(1, *n* = 874) = 23.14, *p* < 0.001]. Moreover, completers more often had a higher partnership satisfaction (*U* = 20215.50, *Z* = −2.15, *p* = 0.032). There were no significant differences between completers and non-completers regarding age, parity, and employment status at T1 (tables on request).

### Mediation Analyses

To analyze whether there is a mediated association between duration of paternal leave and father-infant bonding, two simple mediation analyses were performed. They are presented in Table 3 (without confounders) and Table 4 (controlling for potential confounders). Due to the exclusion of outliers and extreme values as well as some missing data, *n* varied depending on the used variables. The current state of research on mediation analysis states that a significant total effect (*c*, without considering the mediator) is no essential precondition for a mediation analysis. The direct (*c'*) and indirect effects (*ab*) should be interpreted without this preliminary step (Zhao et al., 2010; Rucker et al., 2011).

**Table 3:** Model coefficients for the simple mediation analysis of the association between duration of paternal leave and father-infant bonding through weekly hours spent on childcare without confounders.

		**Consequent**						
		***M*** **(Weekly hours spent on)**				***Y*** **(Father-infant bonding**^**b**^**)**		
		**childcare)**						
**Antecedent**		**Coeff**.	***SE***	***p***		**Coeff**.	***SE***	***p***
*X* (Duration of paternal leave^a^)	*a*	**0.996**	0.369	0.007	*c'*	−0.448	0.233	0.055
*M* (Weekly hours spent on childcare)		—	—	—	*b*	**0.074**	0.029	0.010
Constant	*i*_M_	24.917	0.870	< 0.001	*i*_Y_	111.605	0.909	< 0.001
								
		*R^2^* = 0.015				*R^2^* = 0.020		
		*F*_(1, 511)_ = 7.289, *p* = 0.007				*F*_(2, 510)_ = 4.696, *p* = 0.009		

Simple mediation analysis using ordinary least square regression. Heteroscedasticity consistent standard errors (HC3) employed. N = 513. Outliers and extreme values excluded. X, predictor variable; M, mediator variable; Y, outcome variable; a, effect of X on M; b, effect of M on Y; c', direct effect of X on Y, estimates the difference between X and Y holding M constant; Coeff., unstandardized path coefficients. Significant associations (p < 0.05) are in boldface.

a *Sum of all paternal leave periods in months until T3*.

b *Postpartum Bonding Questionnaire (PBQ); reversed items so that higher scores indicate a higher level of father-infant bonding*.

**Table 4:** Model coefficients for the simple mediation analysis of the association between duration of paternal leave and father-infant bonding through weekly hours spent on childcare including six confounders.

		**Consequent**						
		***M*** **(Weekly hours spent on)**				***Y*** **(Father-infant bonding**^**b**^**)**		
				**childcare)**				
**Antecedent**		**Coeff**.	***SE***	***p***		**Coeff**.	***SE***	***p***
*X* (Duration of paternal leave^a^)	*a*	**1.258**	0.550	0.022	*c'*	**−0.700**	0.353	0.048
*M* (Weekly hours spent on childcare)		—	—	—	*b*	0.059	0.035	0.090
*C*_1_ (Current status of paternal leave)	*f*_1_	**5.306**	1.731	0.002	*g*_1_	0.176	0.934	0.851
*C*_2_ (Part-time work during paternal leave)	*f*_2_	2.470	1.680	0.141	*g*_2_	−1.822	1.151	0.115
*C*_3_ (Duration of solo paternal leave^b^)	*f*_3_	1.380	0.792	0.083	*g*_3_	0.379	0.428	0.377
*C*_4_ (Age)	*f*_4_	**−0.338**	0.132	0.012	*g*_4_	0.122	0.082	0.137
*C*_5_ (Education)	*f*_5_	**−2.717**	1.157	0.019	*g*_5_	−0.666	0.708	0.347
*C*_6_ (Partnership satisfaction^c^)	*f*_6_	−0.096	0.148	0.518	*g*_6_	**0.208**	0.087	0.018
Constant	*i*_M_	37.077	5.356	< 0.001	*i*_Y_	105.218	3.154	< 0.001
								
		*R^2^* = 0.132				*R^2^* = 0.053		
		*F*_(7, 373)_ = 6.158, *p* < 0.001				*F*_(8, 372)_ = 2.073, *p* = 0.038		

*Simple mediation analysis using ordinary least square regression. Heteroscedasticity consistent standard errors (HC3) employed. N = 381. Outliers and extreme values excluded. X, predictor variable; M, mediator variable; Y, outcome variable; C_1−6_, confounders; a, effect of X on M; b, effect of M on Y; c', direct effect of X on Y, estimates the difference between X and Y holding M constant; f_1−6_, effects of C_1−6_ on M; g_1−6_, effects of C_1−6_ on Y; Coeff., unstandardized path coefficients. Significant associations (p < 0.05) are in boldface*.

a *Sum of all paternal leave periods in months until T3*.

b *Sum of all paternal leave periods in months that the father took on his own, i.e., not parallel to maternal leave, until T3*.

c *Short version of the Partnership Questionnaire (“Kurzform des Partnerschaftsfragebogens”, PFB-K)*.

d *Postpartum Bonding Questionnaire (PBQ); reversed items so that higher scores indicate a higher level of father-infant bonding*.

Without considering the confounders, *X* (duration of paternal leave) significantly positively predicted *M* (weekly hours spent on childcare; path *a, B* = 0.996, *p* = 0.007), which in turn significantly positively predicted *Y* (father-infant bonding; path *b, B* = 0.074, *p* = 0.010). The indirect effect of *X* on *Y* was significant, *ab* = 0.073, BCa 95% CI [0.008, 0.167]. The completely standardized indirect effect was 0.034. Considering power, according to a simulation-based calculation of Fritz and MacKinnon (2007), the sample size of this mediation (*n* = 513) was large enough to be able to find even small mediated effects present in the population with sufficient probability.

When considering the confounders, *X* (duration of paternal leave) still significantly positively predicted *M* (weekly hours spent on childcare; path *a, B* = 1.258, *p* = 0.022). However, *M* (weekly hours spent on childcare) did not predict *Y* (father-infant bonding) anymore (path *b*, B = 0.059, *p* = 0.090). Moreover, the indirect effect of *X* on *Y* was not significant anymore, *ab* = 0.075, BCa 95% CI [−0.010, 0.216]. Looking at the direct path, *X* (duration of paternal leave) now significantly negatively predicted *Y* (father-infant bonding; path *c', B* = −0.700, *p* = 0.048). Considering power, the sample size of this adjusted mediation (*n* = 381) was large enough to be able to find combinations of medium-medium as well as medium-large effects on paths *a* and *b* (Fritz and MacKinnon, 2007).

Looking at the individual regression models of the adjusted mediation analysis (Table 4), the individual regression for father-infant bonding (*M* and *C*_1−6_ on *Y*) explained a significant proportion of variance, *R*^2^ = 0.053, *F*_(8, 372)_ = 2.073, *p* = 0.038. The effect size of *f*^2^ = 0.06 was between small and medium. There was only one significant association between the confounders (partnership satisfaction) and *Y* (father-infant bonding). The individual regression model for weekly hours spent on childcare (*X* and *C*_1−6_ on *M*) explained a significant proportion of variance, *R*^2^ = 0.132, *F*_(7, 373)_ = 6.158, *p* < 0.001. The effect size of *f*^2^ = 0.15 was medium. There were some significant associations between the confounders (current status of paternal leave, age, education) and *M* (weekly hours spent on childcare). *Post hoc* power analyses revealed a power of 1.00 for both individual regressions, which was adequate, i.e., above 0.80.

## Discussion

### Summary of Findings

The present study aimed to examine the association between duration of paternal leave and father-infant bonding at 14 months postpartum, potentially mediated by weekly hours spent on childcare. To the best of our knowledge, such a relation had not been researched before. When not considering any confounders, longer duration of paternal leave had a positive effect on father-infant bonding through weekly hours spent on childcare, as hypothesized. However, this indirect path did not stay significant when considering the confounders (current status of paternal leave, part-time work during paternal leave, duration of solo paternal leave, age, education, and partnership satisfaction). Moreover, on the direct path, longer duration of paternal leave now had a negative effect on father-infant bonding. Of the confounders, partnership satisfaction had a positive effect on father-infant bonding.

Factors increasing the number of weekly hours spent on childcare, the mediator, were longer duration of paternal leave, currently being on paternal leave, younger age, and lower educational level. There were no associations between weekly hours spent on childcare and part-time work during paternal leave as well as partnership satisfaction.

### Predictors of Father-Infant Bonding

In this study, we could only find unstable indications that longer periods of paternal leave and more hours spent with the child may strengthen father-infant bonding, suggesting that time might not be its most important facilitator. The underlying mechanisms might be more complex than hypothesized. In female populations, factors which have been repeatedly found to promote mother-infant bonding are factors in close proximity to the birth event, such as a positive birth experience and physical contact in the immediate postpartum period (for overview, see Kinsey and Hupcey, 2013). Those factors might lay an important foundation for father-infant bonding as well and do not take place during paternal leave. Moreover, experiences during paternal leave might not only be positive. Spending a prolonged duration of time with a newborn infant entails challenges and can be demanding, potentially explaining the negative direct effect of paternal leave on father-infant bonding in the adjusted mediation.

Partnership satisfaction was positively related to father-infant bonding, which is in line with previous research (Condon et al., 2013; de Cock et al., 2016; Kerstis et al., 2016; Wynter et al., 2016; Nishigori et al., 2020). Condon et al. (2013) have discussed that this association may be explained by an underlying capacity to form a strong bond or attachment with other human beings. Partnership satisfaction as well as father-infant bonding might represent fathers' attachment behavior or learned attachment schemata. Previous research supports this idea. Multiple studies on father-infant bonding found bonding levels to be stable across different measurement points (Condon et al., 2013; Parfitt et al., 2014; Hall et al., 2015; de Cock et al., 2016). Moreover, fathers who reported to had experienced more care by their own parents—which might facilitate an underlying capacity to form strong bonds (Bretherton, 1987)—showed higher levels of father-infant bonding (Hall et al., 2015).

In view of the above, fathers who do not have the opportunity to take long periods of paternal leave due to employer or financial restrictions still are able to bond with their infant, which is positive. It can reduce pressure for parents to know that pausing work for long periods is not the most important precondition to form a parent-infant bond. Nevertheless, results should not be interpreted in a way that father involvement does not matter for children's development. Father involvement has previously been measured with quantitative (e.g., time, as done in this study) and qualitative (e.g., sensitivity, warmth) measures. Multiple studies have shown positive outcomes of both types of father involvement on child development (for review, see Behson et al., 2018). Only some examples include less externalizing and internalizing problems (Zhang et al., 2019), more prosocial behavior (Flouri, 2008), and increased executive functioning (Meuwissen and Carlson, 2015) of children. While bonding may not be among those, we emphasize that this does not mean that paternal leave is not to be promoted and facilitated.

### Predictors of Weekly Hours Spent on Childcare at 14 Months Postpartum

Some interesting relations were found between the predictors and the mediator, weekly hours spent on childcare. Concerning the specifications of paternal leave, longer periods of paternal leave were found to increase weekly hours spent on childcare at 14 months postpartum. This was expected and in line with previous research (Tanaka and Waldfogel, 2007; Romero-Balsas, 2015; Pragg and Knoester, 2017). Fathers currently being on paternal leave (vs. at work) was the strongest predictor of weekly hours spent on childcare. This indicates that fathers spend the work-free time, which they gain during paternal leave, with their children. If fathers worked part-time during paternal leave, this did not influence their weekly hours spent on childcare at 14 months postpartum. Working part-time during paternal leave might therefore not represent a lesser interest to spend time with the child, but could potentially be a financial necessity for some fathers. Once these fathers complete their paternal leave, they seem to spend just as much of their work-free time with their children as fathers, who had the opportunity to take paternal leave without working part-time. Surprisingly, duration of solo paternal leave did not have a significant impact on weekly hours spent on childcare, which is in line with one prior study examining solo paternal leave (Bünning, 2015). We expected fathers practicing solo paternal leave to be particularly motivated concerning childcare and therefore spend more hours on childcare at 14 months postpartum. However, only a very low percentage of fathers take solo paternal leave, therefore findings should be considered as preliminary.

Older fathers spent less weekly hours on childcare activities such as feeding, putting to bed, dressing, organizing chaperones, playing, or talking at 14 months postpartum. This might reflect the social development that younger German men think fathers should be more involved in childcare (Wippermann, 2017).

Fathers with a higher educational level spent less time on childcare activities at 14 months postpartum. This is in line with a finding by Romero-Balsas (2015), discussing that more educated fathers might have greater work-related responsibilities or reducing work would have greater opportunity costs for them. Contrasting this, Hobler and Pfahl (2015) found that more educated fathers reduce their working hours after the completion of their paternal leave. They argue that more educated fathers can chose their work hours more flexibly. Study results for the influence of education on the duration of paternal leave also vary (Sundström and Duvander, 2002; Lappegard, 2008; Geisler and Kreyenfeld, 2011). In sum, knowledge about the influence of education on father involvement is still limited and non-conclusive. Our results point toward the idea that more educated fathers may have greater work-related responsibilities, such as the expectation to work overtime or business travel, making it more difficult for them to spend time with their child.

Partnership satisfaction did not have a significant influence on weekly hours spent on childcare, which might indicate that fathers do not let their relationship quality influence their motivation to spend time with their child. However, this finding contradicts previous findings (McClain and Brown, 2017; Petts and Knoester, 2019) and the relation might be underestimated in the present study due to systematic dropout of fathers less satisfied.

### Strengths

While research has focused on factors associated with mother-infant bonding (Kinsey and Hupcey, 2013), there are only few studies on father-infant bonding. Our study therefore contributes to a research area that has scarcely been explored and extends the limited existing literature with new information on father-infant bonding and its associated factors. Considering today's fathers wish to be more involved in childcare (Wippermann, 2017; Juncke et al., 2018) and many OECD countries trying to facilitate this (Castro-García and Pazos-Moran, 2016; Gauthier and Bartova, 2018), research addressing fathers' involvement in childcare is highly relevant. As our data were derived from a large population-based cohort study (DREAM; Kress et al., 2019), we were able to include a number of possibly relevant factors. The study combined many specifications of paternal leave (e.g., part-time work during paternal leave or duration of solo paternal leave) as well as fathers' and family aspects (e.g., education or partnership satisfaction).

### Limitations

Some limitations in our analyses need to be addressed. For the present investigation, duration of paternal leave, weekly hours spent on childcare, and father-infant bonding were all measured at the same time (T3). However, duration of paternal leave is a relatively objective information and it can be assumed that paternal leave preceded father-infant bonding, due to the retrospective nature of the question. Concerning time spent on childcare and father-infant bonding however, it can only be spoken of association and not of causation. To be able to meet the assumptions of the linear model, outliers and extreme values were excluded. This led to the final sample of *n* = 637 being smaller in the main analyses and *n* varying significantly between the different analyses. Fathers in our sample took, on average, 2 months of paternal leave, i.e., most fathers took only the two non-transferable partner months. This is in accordance with the general German population (Samtleben et al., 2019). On the one hand, the present findings are therefore generalizable to the German population. On the other hand, the present results cannot infer conclusions regarding populations where more fathers take much longer periods of paternal leave. As for generalizability concerning other study variables, the participants of the present study were predominantly well-educated fathers, which is typical for epidemiological studies (O'Neil, 1979; Søgaard et al., 2004). Additionally, dropout analyses revealed that completers had a higher university degree and partnership satisfaction than non-completers. Considering that education as well as partnership satisfaction were two predictors in the analyses, it is important to be careful generalizing the study's findings to the German population. At the same time, it is important to bear in mind that selection bias does not necessarily influence the results when associations between variables are investigated (Nilsen et al., 2009).

### Future Research Implications

To further elucidate our and previous findings, future research on predictors of father-infant bonding should focus on (a) factors in close proximity to the birth event, such as birth experience and physical contact to the newborn, (b) potentially demanding factors during paternal leave, and (c) father's underlying capacity to bond, for example by considering his own childhood history or his partnership quality. Future research on factors such as mentioned under (a) would benefit from including qualitative measures, for example qualitative assessments of early face-to-face father-infant interactions. As duration of paternal leave and weekly hours spent on childcare predicted father-infant bonding in the unadjusted mediation analysis, research should include these variables as confounders, whenever possible. Moreover, the negative association between duration of paternal leave and father-infant bonding needs to be explored further. In addition, it would be interesting to repeat a similar study in a specific population of fathers with longer durations of paternal leave and solo paternal leave.

Concerning factors predicting weekly hours spent on childcare, there are some uncertainties regarding duration of solo paternal leave, education, and partnership satisfaction. Solo paternal leave of fathers has barely been explored even though its impact should be understood in societies where more and more fathers are actively involved in childcare. Concerning educational level, there are two plausible ideas: Either more educated fathers are hindered to be involved in childcare, due to greater job-related responsibilities (Romero-Balsas, 2015), or more educated fathers have better options to be involved in childcare due to more flexible jobs (Hobler and Pfahl, 2015) and better financial situations. Those ideas should be explored to gain a better understanding of what might help different types of fathers to be more involved in childcare. Moreover, it would be interesting to analyze the impact of education and partnership in a more heterogeneous sample.

### Future Practical Implications

As there was no stable association between duration of paternal leave and father-infant bonding, we cannot conclude that longer periods of paternal leave will strengthen father-infant bonding. Expecting parents could be informed that pausing work for long periods might not be the most important precondition to form a parent-infant bond. This could reduce pressure for parents, who either do not have the opportunity or do not want to take long periods of parental leave. However, we strongly emphasize that paternal involvement is important for many other child outcomes (see section Predictors of Father-Infant Bonding and Behson et al., 2018) and therefore should be promoted and facilitated as currently done by some OECD countries (Castro-García and Pazos-Moran, 2016; Gauthier and Bartova, 2018; Samtleben et al., 2019; Statistisches Bundesamt [Desatis], 2020).

## Conclusion

Since father-infant bonding is crucial for child development (Condon, 1993; Condon and Corkindale, 1998), it is essential to examine and strengthen it. We were especially interested in time as a potential facilitator, as new generations of fathers are spending more time with their children (Wippermann, 2017; Juncke et al., 2018) and OECD countries are facilitating this by passing paternal leave reforms (Castro-García and Pazos-Moran, 2016; Gauthier and Bartova, 2018). The present study drew data of a large population-based cohort study (DREAM; Kress et al., 2019) to examine the association between duration of paternal leave and father-infant bonding at 14 months postpartum, potentially mediated by weekly hours spent on childcare.

Duration of paternal leave positively predicted father-infant bonding through weekly hours spent on childcare. However, this indirect path did not stay significant when considering the confounders (current status of paternal leave, part-time work during paternal leave, duration of solo paternal leave, age, education, and partnership satisfaction). Moreover, in the adjusted model and on the direct path, paternal leave negatively predicted father-infant bonding. These unstable results indicate that the underlying mechanisms might be more complex than hypothesized. Other factors might be more relevant in strengthening father-infant bonding, one of these being partnership satisfaction, which was a significant predictor for father-infant bonding in the present study.

Weekly hours spent on childcare, the mediator, was positively predicted by longer durations of paternal leave and currently being on paternal leave. Age and educational level negatively predicted weekly hours spent on childcare, i.e., younger fathers and fathers with a lower educational level spent more time with their child.

Results suggest that fathers, who do not have the opportunity to take long periods of paternal leave, are still able to form strong bonds with their infants. At the same time, results should not be interpreted in a way that father involvement (e.g., paternal leave/hours spent) does not matter for children's development. Multiple studies have shown other positive outcomes of father involvement, e.g., less externalizing and internalizing problems (Zhang et al., 2019) or more prosocial behavior (Flouri, 2008) of children. The present study's result that longer durations of paternal leave can lead to more father involvement supports the idea that facilitating father involvement can be achieved by paternal leave incentives such as non-transferable father months.

## Data Availability Statement

The datasets presented in this article are not readily available because of legal and ethical constraints. Public sharing of participant data was not included in the informed consent of the study. Requests to access the datasets should be directed to Susan Garthus-Niegel, susan.garthus-niegel@uniklinikum-dresden.de.

## Ethics Statement

The studies involving human participants were reviewed and approved by the Ethics Committee of the Faculty of Medicine of the Technische Universität Dresden (No: EK 278062015). The patients/participants provided their written informed consent to participate in this study.

## Author Contributions

AZ and SG-N conceived the research question. AZ designed and prepared the statistical analyses. RS performed the statistical analyses and drafted the initial manuscript. MK and VK supported the conduction of the study, especially through data collection, and prepared the data for statistical analyses. JM supported the conduction of the study. SG-N acquired the funding, was responsible for conception and design of the basic DREAM study with its sub-studies as well as the coordination and supervision of the data collection and the ongoing cohort study. All authors contributed with the interpretation of the data, contributed to the manuscript revision, read, and approved the final manuscript as submitted and agree to be accountable for all aspects of the work.

## Conflict of Interest

The authors declare that the research was conducted in the absence of any commercial or financial relationships that could be construed as a potential conflict of interest.

## References

[B1] Behson S. Kramer Holmes E. Hill E. J. Robbins N. L. (2018). “Fatherhood, work, and family across the globe: a review and research agenda,” in The Cambridge Handbook of the Global Work-Family Interface, eds. Shockley K. M. Shen W. Johnson R. C. (Cambridge: Cambridge University Press), 614–628.

[B2] Belsky J. (1984). The determinants of parenting: a process model. Child Dev. 55, 83–96. 10.2307/1129836 6705636

[B3] BMFSFJ (2020). Elterngeld, ElterngeldPlus und Elternzeit. Das Bundeselterngeld- und Elternzeitgesetz [Parental allowance, parental allowancePlus and parental leave. The federal parental allowance and parental leave act]. Berlin: Bundesministerium für Familie, Senioren, Frauen und Jugend.

[B4] Brady M. Stevens E. Coles L. Zadoroznyj M. Martin B. (2016). You can spend time. but not necessarily be bonding with them: Australian fathers' constructions and enactments of infant bonding. J. Soc. Policy 46, 1–22. 10.1017/s0047279416000374

[B5] Bretherton I. (1987). “New perspectives on attachment relations: security, communication and internal working models,” in Handbook of Infant Development, ed. J. D. Osofsky (New York, NY: Wiley), 1061–1100.

[B6] Brockington I. F. Fraser C. Wilson D. (2006). The postpartum bonding questionnaire: a validation. Arch. Women's Mental Health 9, 233–242. 10.1007/s00737-006-0132-1 16673041

[B7] Brockington I. F. Oates J. George S. Turner D. Vostanis P. Sullivan M.. (2001). A screening questionnaire for mother-infant bonding disorders. Arch. Women's Mental Health 3, 133–140. 10.1007/s007370170010

[B8] Bünning M. (2015). What happens after the “daddy months”? Fathers' involvement in paid work, childcare, and housework after taking parental leave in Germany. Eur. Sociol. Rev. 31, 738–748. 10.1093/esr/jcv072

[B9] Castro-García C. Pazos-Moran M. (2016). Parental leave policy and gender equality in Europe. Fem. Econ. 22, 51–73. 10.1080/13545701.2015.1082033

[B10] Condon J. Corkindale C. Boyce P. Gamble E. (2013). A longitudinal study of father-to-infant attachment: antecedents and correlates. J. Reprod. Infant Psychol. 31, 15–30. 10.1080/02646838.2012.757694

[B11] Condon J. T. (1993). The assessment of antenatal emotional attachment: development of a questionnaire instrument. Br. J. Med. Psychol. 66, 167–183. 10.1111/j.2044-8341.1993.tb01739.x 8353110

[B12] Condon J. T. Corkindale C.J. (1998). The assessment of parent-to-infant attachment: development of a self-report questionnaire instrument. J. Reprod. Infant Psychol. 16, 57–76. 10.1080/02646839808404558

[B13] Davidson R. MacKinnon J. G. (1993). Estimation and Inference in Econometrics. New York, NY: Oxford University Press.

[B14] de Cock E. S. A. Henrichs J. Klimstra T. A. Maas A. J. B. M. Vreeswijk C. M. J. M. Meeus W. H. J.. (2017). Longitudinal associations between parental bonding, parenting stress, and executive functioning in toddlerhood. J. Child Family Stud. 26, 1723–1733. 10.1007/s10826-017-0679-7 28572718 PMC5429904

[B15] de Cock E. S. A. Henrichs J. Vreeswijk C. M. J. M. Maas A. J. B. M. Rijk C. H. A. M. van Bakel H. J. A. (2016). Continuous feelings of love? The parental bond from pregnancy to toddlerhood. J. Family Psychol. 30, 125–134. 10.1037/fam0000138 26280095

[B16] Faul F. Erdfelder E. Lang A.-G. Buchner A. (2007). G^*^Power 3: a flexible atatistical power analysis program for the social, behavioral, and biomedical sciences. Behav. Res. Methods 39, 175–191. 10.3758/bf03193146 17695343

[B17] Flouri E. (2008). Fathering and adolescents' psychological adjustment: the role of fathers' involvement, residence and biology status. Child. Care. Health Dev. 34, 152–161. 10.1111/j.1365-2214.2007.00752.x 18257787

[B18] Fritz M. S. MacKinnon D. P. (2007). Required sample size to detect the mediated effect. Psychol. Sci. 18, 233–239. 10.1111/j.1467-9280.2007.01882.x 17444920 PMC2843527

[B19] Fuchs A. Möhler E. Reck C. Resch F. Kaess M. (2016). The early mother-to-child bond and its unique prospective contribution to child behavior evaluated by mothers and teachers. Psychopathology 49, 211–216. 10.1159/000445439 27383771

[B20] Gauthier A. H. Bartova A. (2018). “The impact of leave policies on employment, fertility, gender equality, and health,” in The Cambridge Handbook of the Global Work-Family Interface, eds Shockley K. M. Shen W. Johnson R. C. (Cambridge: Cambridge University Press), 120–137.

[B21] Geisler E. Kreyenfeld M. (2011). Against all odds: fathers' use of parental leave in Germany. J. Eur. Soc. Policy 21, 88–99. 10.1177/0958928710385732

[B22] German National Cohort Consortium (2014). The german national cohort: aims, study design and organization. Eur. J. Epidemiol. 29, 371–382. 10.1007/s10654-014-9890-7 24840228 PMC4050302

[B23] Hall R. A. Hoffenkamp H. N. Tooten A. Braeken J. Vingerhoets A. J. Van Bakel H. J. (2015). Child-rearing history and emotional bonding in parents of preterm and full-term infants. J. Child Family Stud. 24, 1715–1726. 10.1007/s10826-014-9975-7

[B24] Hall S. S. MacDermid S. M. (2009). A typology of dual earner marriages based on work and family arrangements. J. Fam. Econ. Issues 30, 215–225. 10.1007/s10834-009-9156-9

[B25] Harris P. A. Taylor R. Minor B. L. Elliott V. Fernandez M. O'Neal L.. (2019). The REDCap consortium: building an international community of software platform partners. J. Biomed. Inform. 95:103208. 10.1016/j.jbi.2019.103208 31078660 PMC7254481

[B26] Harris P. A. Taylor R. Thielke R. Payne J. Gonzalez N. Conde J. G. (2009). Research electronic data capture (REDCap) – a metadata-driven methodology and workflow process for providing translational research informatics support. J. Biomed. Inform. 42, 377–381. 10.1016/j.jbi.2008.08.010 18929686 PMC2700030

[B27] Hayes A.F. (2018). Introduction to Mediation, Moderation, and Conditional Process Analysis: A Regression-Based Approach. New York, NY: The Guilford Press.

[B28] Hobler D. Pfahl S. (2015). Einflussfaktoren auf die Arbeitszeitdauer von Vätern nach den Elterngeldmonaten [Factors Influencing the Duration of Fathers' Working Time after the Parental Allowance Months]. Berlin: Friedrich-Ebert-Stiftung.

[B29] IBM Corp (2019). IBM SPSS Statistics for Windows. 26.0 ed (Armonk, NY: IBM Corp.).

[B30] Jessee V. Adamsons K. (2018). Father involvement and father-child relationship quality: an intergenerational perspective. Parent. Sci. Pract. 18, 28–44. 10.1080/15295192.2018.1405700 30881229 PMC6415916

[B31] Juncke D. Braukmann J. Heimer A. (2018). Väterreport. Vater sein in Deutschland heute [Fathers' Report. Being a Father in Germany Today]. Berlin: Bundesministerium für Familie, Senioren, Frauen und Jugend.

[B32] Kerstis B. Aarts C. Tillman C. Persson H. Engström G. Edlund B.. (2016). Association between parental depressive symptoms and impaired bonding with the infant. Arch. Women's Mental Health 19, 87–94. 10.1007/s00737-015-0522-3 25854998

[B33] Kinsey C. B. Hupcey J. E. (2013). State of the science of maternal-infant bonding: a principle-based concept analysis. Midwifery 29, 1314–1320. 10.1016/j.midw.2012.12.019 23452661 PMC3838467

[B34] Kliem S. Job A. K. Kröger C. Bodenmann G. Stöbel-Richter Y. Hahlweg K.. (2012). Entwicklung und Normierung einer Kurzform des Partnerschaftsfragebogens (PFB-K) an einer repräsentativen deutschen Stichprobe [Development and standardisation of a short form of the Partnership Questionnaire (PFB-K) on a representative German sample]. Zeitschrift für Klinische Psychol. Psychother. 41, 81–89. 10.1026/1616-3443/a000135

[B35] Kress V. Steudte-Schmiedgen S. Kopp M. Förster A. Altus C. Schier C.. (2019). The impact of parental role distributions, work participation, and stress factors on family health-related outcomes: study protocol of the prospective multi-method cohort “Dresden study on parenting, work, and mental health” (DREAM). Front. Psychol. 10:1273. 10.3389/fpsyg.2019.01273 31263435 PMC6584823

[B36] Lappegard T. (2008). Changing the gender balance in caring: fatherhood and the division of parental leave in Norway. Popul. Res. Policy Rev. 27, 139–159. 10.1007/s11113-007-9057-2

[B37] Mason Z. S. Briggs R. D. Silver E. J. (2011). Maternal attachment feelings mediate between maternal reports of depression, infant social-emotional development, and parenting stress. J. Reprod. Infant Psychol. 29, 382–394. 10.1080/02646838.2011.629994

[B38] McClain L. Brown S. L. (2017). The roles of fathers' involvement and coparenting in relationship quality among cohabiting and married parents. Sex Roles 76, 334–345. 10.1007/s11199-016-0612-3 30555203 PMC6294450

[B39] Meuwissen A. S. Carlson S. M. (2015). Fathers matter: the role of father parenting in preschoolers' executive function development. J. Exp. Child Psychol. 140, 1–15. 10.1016/j.jecp.2015.06.010 26209884 PMC4558369

[B40] Nilsen R. M. Vollset S. E. Gjessing H. K. Skjaerven R. Melve K. K. Schreuder P.. (2009). Self-selection and bias in a large prospective pregnancy cohort in Norway. Paediatr. Perinat. Epidemiol. 23, 597–608. 10.1111/j.1365-3016.2009.01062.x 19840297

[B41] Nishigori H. Obara T. Nishigori T. Metoki H. Mizuno S. Ishikuro M.. (2020). Mother-to-infant bonding failure and intimate partner violence during pregnancy as risk factors for father-to-infant bonding failure at 1 month postpartum: an adjunct study of the Japan Environment and Children's Study. J. Maternal-Fetal Neonatal Med. 33, 2789–2796. 10.1080/14767058.2018.1560414 30563397

[B42] O'Neil M.J. (1979). Estimating the nonresponse bias due to refusals in telephone surveys. Public Opin. Q. 43, 218–232. 10.1086/268513

[B43] Parfitt Y. Ayers S. Pike A. Jessop D. Ford E. (2014). A prospective study of the parent-baby bond in men and women 15 months after birth. J. Reprod. Infant Psychol. 32, 441–456. 10.1080/02646838.2014.956301

[B44] Petts R. J. Knoester C. (2019). Paternity leave and parental relationships: variations by gender and mothers' work statuses. J. Marriage Family 81, 468–486. 10.1111/jomf.12545 30858623 PMC6407703

[B45] Petts R. J. Knoester C. Waldfogel J. (2020). Fathers' paternity leave-taking and children's perceptions of father-child relationships in the United States. Sex Roles 82, 173–188. 10.1007/s11199-019-01050-y 32076360 PMC7030161

[B46] Pfahl S. Reuyss S. (2010). “Das neue Elterngeld: Erfahrungen und betriebliche Nutzungsbedingungen von Vätern: Eine explorative Studie [The new parental allowance: experiences and operational usage conditions of fathers: an exploratory study],” in Vielfalt managen: Gesundheit fördern – Potenziale nutzen [Managing Diversity: Promoting Health – Harnessing Potential], eds Badura B. Schröder H. Klose J. Macco K. (Berlin: Springer), 225–233.

[B47] Pragg B. Knoester C. (2017). Parental leave use among disadvantaged fathers. J. Fam. Issues 38, 1157–1185. 10.1177/0192513X15623585 28694555 PMC5501417

[B48] Reck C. Klier C. M. Pabst K. Stehle E. Steffenelli U. Struben K.. (2006). The German version of the postpartum bonding instrument: psychometric properties and association with postpartum depression. Arch. Women's Mental Health 9, 265–271. 10.1007/s00737-006-0144-x 16937316

[B49] Romero-Balsas P. (2015). Consequences of paternity leave on allocation of childcare and domestic tasks. Rev. Española Investigaciones Sociol. 149, 87–109. 10.5477/cis/reis.149.87

[B50] Rucker D. D. Preacher K. J. Tormala Z. L. Petty R. E. (2011). Mediation analysis in social psychology: current practices and new recommendations. Soc. Personal. Psychol. Compass 5, 359–371. 10.1111/j.1751-9004.2011.00355.x

[B51] Samtleben C. Schäper C. Wrohlich K. (2019). Elterngeld und Elterngeld Plus: Nutzung durch Väter gestiegen, Aufteilung zwischen Müttern und Vätern aber noch sehr ungleich [Parental allowance and parental allowancePlus: use by fathers increased, but distribution between mothers and fathers still very unequal]. Berlin: Deutsches Institut für Wirtschaftsforschung e.V.

[B52] Scism A. R. Cobb R. L. (2017). Integrative review of factors and interventions that influence early father-infant bonding. J. Obstetr. Gynecol. Neonatal Nursing 46, 163–170. 10.1016/j.jogn.2016.09.004 28061325

[B53] Søgaard A. J. Selmer R. Bjertness E. Thelle D. (2004). The Oslo health study: the impact of self-selection in a large, population-based survey. Int. J. Equity Health 3, 1–12. 10.1186/1475-9276-3-3 15128460 PMC428581

[B54] Statistisches Bundesamt [Desatis] (2020). Entwicklung der Väterbeteiligung für ab dem Jahr 2008 geborene Kinder nach Ländern [Development of fathers' participation; children born after 2008; by country] [Online]. Wiesbaden: Statistisches Bundesamt. Available online at: https://www.destatis.de/DE/Themen/Gesellschaft-Umwelt/Soziales/Elterngeld/Tabellen/zeitreihe-elterngeld.html (accessed February 3, 2021).

[B55] Sundström M. Duvander A.-Z. E. (2002). Gender division of childcare and the sharing of parental leave among new parents in Sweden. Eur. Sociol. Rev. 18, 433–447. 10.1093/esr/18.4.433

[B56] Tanaka S. Waldfogel J. (2007). Effects of parental leave and work hours on fathers' involvement with their babies. Commun. Work Family 10, 409–426. 10.1080/13668800701575069

[B57] Wilson K. R. Prior M. R. (2010). Father involvement: the importance of paternal solo care. Early Child Dev. Care 180, 1391–1405. 10.1080/03004430903172335

[B58] Wippermann C. (2017). Männer-Perspektiven. Auf dem Weg zu mehr Gleichstellung? [Men's Perspectives. On the Way to more Equality?]. Berlin: Bundesministerium für Familie, Senioren, Frauen und Jugend.

[B59] Wynter K. Rowe H. Tran T. Fisher J. (2016). Factors associated with father-to-infant attachment at 6 months postpartum: a community-based study in Victoria, Australia. J. Reproduct. Infant Psychol. 34, 185–195. 10.1080/02646838.2015.1136051

[B60] Yalçin S. S. Örün E. Mutlu B. Madendag Y. Sinici I. Dursun A.. (2010). Why are they having infant colic? A nested case-control study. Paediatr. Perinatal Epidemiol. 24, 584–596. 10.1111/j.1365-3016.2010.01150.x 20955236

[B61] Zhang J. Liu Y. Hu T. (2019). A meta-analysis of the relationship between father involvement and problem behaviour among preschool children. Early Child Dev. Care 27, 1–23. 10.1080/03004430.2019.1679127

[B62] Zhao X. Lynch J. G. Chen Q. (2010). Reconsidering Baron and Kenny: myths and truths about mediation analysis. J. Consumer Res. 37, 197–206. 10.1086/651257

